# Obesity, High-Molecular-Weight (HMW) Adiponectin, and Metabolic Risk Factors: Prevalence and Gender-Specific Associations in Estonia

**DOI:** 10.1371/journal.pone.0073273

**Published:** 2013-09-09

**Authors:** Triin Eglit, Inge Ringmets, Margus Lember

**Affiliations:** 1 Department of Internal Medicine, University of Tartu and Tartu University Hospital, Tartu, Estonia; 2 Department of Public Health, University of Tartu, Tartu, Estonia; University College London, United Kingdom

## Abstract

**Background:**

The metabolic consequences of obesity are associated with an imbalance of adipocytokines, e.g. adiponectin. However, some obese subjects remain metabolically healthy and have adiponectin levels similar to normal body weight subjects. Current estimates of the prevalence of obesity in Estonia have relied only on self-report data.

**Objectives:**

To estimate the prevalence of obesity in Estonia, to test for associations between HMW adiponectin and metabolic risk factors and to test if HMW adiponectin levels differentiate metabolically healthy and metabolically unhealthy subjects.

**Methods:**

We conducted a population-based cross-sectional multicentre study to gather history, examination and blood test results for 495 subjects aged 20–74. Metabolically healthy subjects were free from hypertension, dyslipidaemia, impaired glucose regulation and insulin resistance. Metabolically unhealthy subjects had at least one of these four metabolic abnormalities.

**Results:**

The prevalence of obesity was 29% in men and 34% in women. HMW adiponectin was positively correlated with HDL cholesterol and negatively correlated with triglycerides, obesity, insulin resistance and blood glucose. This effect was driven by metabolically unhealthy subjects in men, but by both metabolically healthy and metabolically unhealthy subjects in women. Metabolically healthy women had higher HMW adiponectin levels than metabolically unhealthy women. 12% of all obese subjects were metabolically healthy, and their HMW adiponectin levels were similar to normal weight subjects.

**Conclusions:**

Obesity is more prevalent in Estonian adults than previously thought. HMW adiponectin levels were associated with various metabolic risk factors in metabolically healthy women but not in metabolically healthy men. For both genders, HMW adiponectin differentiates metabolically healthy obese subjects from metabolically unhealthy obese subjects.

## Introduction

Obesity is recognized as the most prevalent metabolic disease world-wide, reaching epidemic proportions in both developed and developing countries. The prevalence of obesity in Europe ranges between 10−25% for men and 10−30% for women [1]. To date, the prevalence of obesity in Estonia has only been estimated from self-reported data from posted questionnaire surveys. No population-based study using objective measurements of weight and height has been conducted in Estonia.

Being overweight or obese causes and exacerbates a large number of health problems, both independently and in association with other risk factors and diseases [2]. This relationship between obesity and subsequent disease is thought to be mediated by the metabolic changes of obesity. Adipocytes and adipose tissue produce a wide range of hormones and cytokines [3], whereby imbalances in pro- and anti-inflammatory adipocytokines have been associated to the metabolic consequences of obesity [4]. Plasma adipocytokine levels rise with an increase in adipose tissue and adipocyte volume, except for plasma adiponectin which is lower in obesity. Being solely produced by adipocytes, a low plasma adiponectin concentration is a good measure of adipocyte dysfunction [3]. Adiponectin is termed a “beneficial” adipocytokine due to its anti-inflammatory, anti-atherogenic, anti-diabetic and cardioprotective effects and promotion of good endothelial function [5]. Multiple studies have indicated that the high-molecular-weight (HMW) form of adiponectin is its most active form [3], [5], [6]. Serum HMW adiponectin values are inversely correlated with the presence of metabolic syndrome in both genders [6], [7]. Analysis of the relationship between HMW adiponectin and components of metabolic syndrome have shown that HMW adiponectin is inversely associated with triglycerides, obesity and fasting glucose, and positively associated with HDL cholesterol [9]–[12]. There is clear gender difference in HMW adiponectin levels: women have significantly higher HMW adiponectin levels compared to men [7], [8]. The relationship between HMW adiponectin and blood pressure has varied between different studies and also by gender [9]–[12]. This calls for more detailed subtype analyses of the association between adiponectin, gender and metabolic risk factors.

Interestingly, not all obese individuals develop the metabolic and cardiovascular disorders associated with obesity. It has been hypothesised that this is due to the preservation of normal adipose tissue architecture and function [13]. While no uniform definition exists for any phenotypical subtypes of obesity, the term “metabolically normal obesity” describes the absence of any overt cardiometabolic disease [14]. Several studies have shown that such a metabolically healthy obese phenotype does exist [15] and is characterised beside other factors by adiponectin levels similar to normal body weight subjects [16], [17]. However, data about HMW adiponectin levels in metabolically healthy obese phenotype is scarce. To the best of our knowledge, only few studies exist that estimated serum HMW adiponectin levels in obese but metabolically healthy women [18], [19].

**The aims** **of our study** **were:** to estimate the prevalence of overweight and obesity in the Estonian adult population; to test for correlations between HMW adiponectin and metabolic risk factors; and to compare HMW adiponectin levels between metabolically healthy and unhealthy subjects.

## Methods

A population-based, cross-sectional, multicentre study was conducted between November 2008 and May 2009 in three different counties of Estonia, to establish the prevalence of metabolic disorders and associated risk factors. The study population consisted of randomly selected adults, aged 20 to 74 years, from four general practices. Study participants were representative of the general Estonian population in terms of age and gender. An invitation letter about the study was sent to each participant. The total response rate was 53.2 percent, resulting in a total study population of 495 subjects.

On the day of the study, subjects visited their GPs in the morning between 8 a.m. and 11 a.m. after an overnight fast (lasting at least 10 hours). An informed consent form was signed, and blood pressure, waist circumference, height, and weight were measured with participants wearing their indoor clothes without shoes. Blood pressure was measured using a mercury sphygmomanometer after the patient had been sitting for at least five minutes. The mean of three consecutive measurements was used for analysis, with at least a three minute interval between each measurement. A face-to-face clinical interview was conducted to assess for the presence of other medical conditions and cardiovascular risk factors. A standard oral glucose tolerance test (WHO 1999) was conducted with Glycodyn^®^ solution (Biofile Ltd, Turku, Finland), except for those subjects with known diabetes mellitus. Plasma glucose was measured by the hexokinase method. Total cholesterol, HDL cholesterol, and triglycerides were measured using an enzymatic colorimetric assay (COBAS INTEGRA 800 plus analyzer, Roche, Basel, Switzerland). Plasma insulin was measured using a chemiluminescent assay (Immulite 2000 analyzer, Siemens Healthcare Diagnostics, Deerfield, IL, USA). Plasma-fasting HMW adiponectin was measured subsequently in 458 subjects (191 men) from plasma samples that had been stored at −80°C for a maximum of 2.5 years and had never been thawed. HMW adiponectin was detected by Adiponectin (Multimetric) ELISA (ALPCO Diagnostics, Salem, NH, USA), using an automatic ELISA Triturus analyzer (Grifols International, Barcelona, Spain). The intra-assay coefficient of variation for the HMW adiponectin ELISA was 4.8 percent (n = 9), and the interassay coefficient of variation was 6.9 percent (n = 7).

Subjects were categorized as normal weight (BMI 18.5−24.9 kg/m^2^), overweight (BMI 25.0–29.9 kg/m^2^), and obese (BMI ≥30.0 kg/m^2^) [20]. Impaired glucose regulation was diagnosed according to WHO criteria [21]. Insulin resistance (IR) was estimated using the homeostasis model assessment (HOMA): HOMA-IR  =  fasting glucose (mmol/l) x fasting insulin (mU/l)/22.5 [22]. IR was defined as the upper quartile of HOMA-IR in the whole study group (exempting subjects with previously known diabetes mellitus). The threshold for the whole study group was 1.92 (2.04 and 1.82 for men and women, respectively).

Metabolically healthy subjects were defined as subjects without impaired glucose regulation, without dyslipidaemia (triglycerides ≥1.7 mmol/L or HDL cholesterol <1.3 mmol/L in women or HDL cholesterol <1.03 mmol/l in men or lipid-lowering treatment) and without hypertension (blood pressure ≥130/80 mmHg or on antihypertensive treatment) and without insulin resistance. “Metabolically unhealthy” was defined as having at least one of the above-mentioned four risk factors. The study was approved by the University of Tartu Ethics Review Committee on Human Research.

### Statistical analysis

Due to the slight underrepresentation of younger age-groups (20–39 years), the prevalence of obesity was weighted to the Estonian 20−74 year old population (estimated in 2009). The prevalence of obesity was presented as a proportion with 95% confidence intervals (95% CI-s). The chi-square test (with Bonferroni correction) was used for multiple comparison of prevalence between three age-groups. Descriptive statistics as medians and interquartile ranges or as means and standard deviations were calculated for the continuous variables. The Mann-Whitney U test was used for comparisons between different groups. Spearman partial correlations coefficients were used to estimate for associations between HMW adiponectin and metabolic risk factors. P values were considered statistically significant at the 0.05 level. Statistical analysis was performed using R software version 2.15.3 [23].

## Results

Demographic data about the study subjects has been published previously [7].

### Prevalence of overweight and obesity

The prevalence of obesity (BMI≥30) in the whole study group was 34% (31% for men and 36% for women). The prevalence of obesity weighted for the Estonian population in 2009 was 32% (29% for men and 34% for women). The prevalence of obesity was significantly higher in the oldest age-group compared with the youngest age group: 48% vs. 27%, respectively, p = 0.0002. There was no significant gender difference in prevalence of obesity (Table 1). The prevalence of being overweight or obese (defined as BMI ≥25) was 67% (95% CI 62−71) in the study group, with 72% (95% CI 66−78) for men and 63% (95% CI 57−68) for women.

**Table 1:** The age- and gender-specific prevalence of obesity in Estonian adult population.

Age-group	N	% (95% CI)
**20−44 years (n = 221)**	**59**	**26.7 (21.1−33.1)**
Men (n = 105)	29	27.6 (19.6−37.3)
Women (n = 116)	30	25.9 (18.4−35.0)
**45−60 years (n = 162)**	**55**	**34.0 (26.8−41.9)**
Men (n = 64)	17	26.6 (16.7−39.3)
Women (n = 98)	38	38.8 (29.3−49.2)
**61−74 years (n = 111)**	**53**	**47.7 (38.3−57.4)**
Men (n = 45)	20	44.4 (30.0−59.9)
Women (n = 66)	33	50.0 (38.3−61.7)
**Total (n = 494)**	**167**	**33.8 (29.7−38.2)**
*** Weighted***		***31.8 (26.9−36.7)***
Men (n = 214)	66	30.8 (24.8−37.6)
* Weighted*		*29.1 (21.9−36.2)*
Women (n = 280)	101	36.1 (30.5−42.0)
* Weighted*		*34.1 (27.4−40.8)*

### Correlations between HMW adiponectin and metabolic risk factors

We assessed for correlations between HMW adiponectin and metabolic risk factors in the whole study group after adjusting for age. In both genders, HMW adiponectin was positively correlated with HDL cholesterol, and negatively correlated with triglycerides, waist circumference, BMI, HOMA-IR, fasting insulin and fasting glucose. For women only, a significant negative correlation was found between HMW adiponectin and systolic and diastolic blood pressure as well as 2 h glucose levels on the oral glucose tolerance test (OGTT) (Table 2).

**Table 2:** Spearman partial correlation coefficients (adjusted for age) between HMW adiponectin and various metabolic risk factors in metabolically healthy and unhealthy subjects.

	Men			Women		
Risk factors	Metabolically healthy n = 52	Metabolically unhealthy n = 139	Whole group n = 191	Metabolically healthy n = 99	Metabolically unhealthy n = 168	Whole group n = 267
HDL cholesterol	0.27	0.39***	0.38***	0.32**	0.51***	0.55***
Triglycerides	−0.07	−0.36***	−0.33***	−0.25**	−0.32***	−0.39***
Waist circumference	−0.09	−0.30**	−0.29***	−0.16	−0.34***	−0.38***
BMI	−0.18	−0.38***	−0.34***	−0.14	−0.28**	−0.33***
HOMA-IR	−0.22	−0.44***	−0.40***	−0.27**	−0.38***	−0.42***
Fasting insulin	−0.21	−0.44***	−0.40***	−0.31**	−0.35***	−0.40***
Fasting glucose	−0.08	−0.15	−0.16*	−0.03	−0.27**	−0.28***
2h glucose in OGTT	−0.1	−0.07	−0.05	−0.17°	−0.20*	−0.27***
Systolic blood pressure	0.32*	0.06	0.05	0.04	−0.19*	−0.26***
Diastolic blood pressure	0.2	0.0006	−0.02	−0.10	−0.09	−0.23**

P<0.05 presented „bold“,°p<0.1; *p<0.05; **p<0.01; ***p<0.001

HDL – high-density lipoprotein, BMI– body mass index, HOMA-IR – homeostasis model of insulin resistance, OGTT – oral glucose tolerance test.

**Healthy** – subjects without impaired glucose regulation, hypertension (blood pressure ≥130/80 mmHg), dyslipidaemia (triglycerides ≥1.7 mmol/L or HDL<1.03 in men or HDL<1.3 in women) and insulin resistance.

**Unhealthy** – subjects with impaired glucose regulation and/or hypertension and/or dyslipidaemia and/or insulin resistance.

**Insulin resistance** – was defined as the upper quartile of HOMA-IR in the whole study group (exempting subjects with previously known diabetes mellitus), the threshold of which in whole study group was found to be 1.92 (2.04 and 1.82 for men and women, resp.).

### Comparison of HMW adiponectin and metabolic risk factors between metabolically healthy and unhealthy subjects

Among the whole study group, 33% of subjects were metabolically healthy (151/458 subjects, whereby prevalence was 27% in men and 37% in women, *p*-value = 0.03). Among obese subjects, 12% of subjects were metabolically healthy (19/158 subjects, whereby prevalence was 11% in men (6/57 subjects) and 13% in women (13/101 subjects), *p*-value = 0.9). The percentages of the study subjects who used antihypertensive, lipid-lowering and antidiabetic treatment were 26.6, 3.9 and 4.1%, respectively and there was no gender difference. We did not find significant associations between HMW adiponectin level and smoking and between HMW adiponectin level and alcohol consumption.

We then asked “Among both obese and non-obese subjects alike, is this correlation between HMW adiponectin and metabolic risk factors driven largely by data from the metabolically *healthy* subjects, or by data from metabolically *unhealthy* subjects”? We assessed for effects in men and women separately, and uncovered gender-specific differences (Table 2). In men, the correlation between HMW adiponectin and metabolic risk factors is driven exclusively by the metabolically unhealthy subgroup. In women too, the metabolically unhealthy subgroup again drives the majority of this correlation. However, we also found metabolically healthy women to show correlations between HMW adiponectin and HDL cholesterol, triglycerides and insulin resistance. (Table 2). Moreover, metabolically healthy women showed substantially higher HMW adiponectin levels compared with metabolically unhealthy women. In men we did not found significant difference in median HMW adiponectin levels between metabolically healthy and unhealthy subjects. Metabolically healthy subjects were significantly younger and had significantly lower HOMA-IR, waist circumference and BMI compared with metabolically unhealthy subjects in both genders (Table 3).

**Table 3:** Comparison of metabolically healthy and unhealthy subjects.

	Men			Women		
Characteristics	Healthy n = 52	Unhealthy n = 139	p-value	Healthy n = 99	Unhealthy n = 168	p-value
HMW adiponectin (μg/ml)*	2.732 (1.784−3.683)	2.436 (1.442−3.941)	0.4	5.357 (3.606−6.722)	4.146 (2.421−5.795)	**0.002**
HOMA-IR*	0.525 (0.440−0.908)	1.510 (0.760−2.760)	**0.0001**	0.670 (0.450−1.105)	1.560 (0.885−2.715)	**0.0001**
Waist circumference (cm) °	89.1±11.2	100.9±13.1	**0.0001**	81.1±11.1	95.9±16.1	**0.0001**
BMI (kg/m^2^) °	25.1±4.5	28.9±5.1	**0.0001**	24.8±4.4	30.8±6.9	**0.0001**
Age (years) °	38.2±13.1	50.9±13.5	**0.0001**	41.3±13.1	53.5±13.7	**0.0001**

Data presented as median (interquartile range) * or mean (±SD) °.

**Healthy** – subjects without impaired glucose regulation, hypertension (blood pressure ≥130/80 mmHg), dyslipidaemia (triglycerides ≥1.7 mmol/L or HDL<1.03 in men or HDL<1.3 in women) and insulin resistance.

**Unhealthy** – subjects with impaired glucose regulation and/or hypertension and/or dyslipidaemia and/or insulin resistance.

**Insulin resistance** – was defined as the upper quartile of HOMA-IR in the whole study group (exempting subjects with previously known diabetes mellitus), the threshold of which in whole study group was found to be 1.92 (2.04 and 1.82 for men and women, resp.).

### Comparison of HMW adiponectin levels between metabolically healthy Vs unhealthy overweight/obese subjects

Among overweight or obese subjects (BMI≥25), we compared metabolically healthy and unhealthy subjects. In both genders, metabolically healthy subjects were significantly younger and had significantly smaller waist circumference and lower HOMA-IR (Table 4). The HOMA-IR level in metabolically healthy overweight/obese subjects was comparable to those with a normal BMI. Among women, metabolically healthy overweight/obese subjects had higher HMW adiponectin levels compared with metabolically unhealthy overweigh/obese women. The HMW adiponectin levels of metabolically healthy overweight/obese women were comparable to those women with a normal BMI (Table 4). To summarize, adiponectin is only useful for women, in differentiating metabolically healthy overweight/obese subjects from metabolically unhealthy overweight/obese subjects.

**Table 4:** Comparison of metabolically healthy and metabolically unhealthy subjects in terms of HMW adiponectin, HOMA-IR, age and waist circumference.

	BMI 18.5–24.9	BMI≥25 Healthy	BMI≥25 Unhealthy	p-value Healthy/ Unhealthy	BMI≥30 Healthy	BMI≥30 Unhealthy	p-value Healthy/ Unhealthy
**HMW adiponectin** (μg/ml)*							
Men	3.297 n = 53	2.266 n = 27	2.163 n = 110	0.9	3.338 n = 6	1.957 n = 51	**0.04**
Women	5.151 n = 91	5.099 n = 39	3.824 n = 132	**0.02**	5.079 n = 13	3.091 n = 88	0.1
**HOMA-IR**°							
Men	0.80±0.92	0.86±0.44	2.44±2.09	**0.0001**	1.08±0.61	3.15±2.39	**0.006**
Women	0.92±0.73	0.94±0.45	2.14±1.60	**0.0001**	1.14±0.42	2.53±1.71	**0.0009**
**Age (years)** °							
Men	40.3±14.4	43.4±13.9	52.1±12.8	**0.006**	54.8±9.6	52.5±13.0	0.7
Women	43.3±14.6	44.5±12.4	54.7±12.8	**0.0001**	48.4±9.9	53.6±13.2	0.1
**Waist circum-ference (cm)** °							
Men	84.1±9.1	97.1±9.2	104.6±11.1	**0.0008**	110.8±5.6	113.4±8.8	0.5
Women	75.0±6.4	91.6±8.0	101.2±13.7	**0.0001**	100.1±6.0	107.4±11.8	**0.01**

Data presented as median * or mean (±SD) °.

**Healthy** – subjects without impaired glucose regulation, hypertension (blood pressure ≥130/80 mmHg), dyslipidaemia (triglycerides ≥1.7 mmol/L or HDL<1.03 in men or HDL<1.3 in women) and insulin resistance.

**Unhealthy** – subjects with impaired glucose regulation and/or hypertension and/or dyslipidaemia and/or insulin resistance. **Insulin resistance** – was defined as the upper quartile of HOMA-IR in the whole study group (exempting subjects with previously known diabetes mellitus), the threshold of which in whole study group was found to be 1.92 (2.04 and 1.82 for men and women, resp.)

When looking at only obese subjects, median HMW adiponectin levels between metabolically healthy and metabolically unhealthy subjects were 3.338 vs 1.957 (p = 0.04) for men, and 5.079 vs 3.091 μg/ml (p = 0.1) for women. This suggests that for obese subjects of both genders, metabolically healthy subjects have HMW adiponectin levels that are comparable to subjects in normal weight. These differences may become more significant with larger sample sizes. Metabolically healthy obese subjects were significantly less insulin resistant than metabolically unhealthy obese subjects in both genders. Interestingly, metabolically healthy and metabolically unhealthy obese subjects did not differ by mean age. (Table 4).

## Discussion

### Prevalence of overweight and obesity

In this population-based, multicentre, cross-sectional study we found that the prevalence of obesity in Estonian adults aged 20−74 was significantly higher than previously estimated: 32% (29% in men and 34% in women, weighted for the Estonian population in 2009). In comparison, the prevalence estimated by self-reported data in 2008 among subjects aged 16–64 was just 17.5% for men and 18.0% for women [24]. The prevalence of obesity increases with age and our study group was slightly older. However, this slight shift in demographics is insufficient to explain this nearly twofold difference. Previous studies have shown that the prevalence of being overweight is higher if calculated from measured values, as opposed to self-reported values [25]. Furthermore, non-responders to self-reported body weight questions in health questionnaires are more likely to be obese [26]. These factors may partially explain why self-report methods underestimate the prevalence of obesity.

Recent European studies using objective data have assessed the prevalence of obesity in the same magnitude what we currently found in Estonia: for example in Latvia 26% of men and 33% of women [27], in Albania 24% of men and 36% of women [28] and in Croatia 26% of men and 34% of women [29]. The high prevalence of overweight and obesity (defined as BMI ≥25) in Estonia (67%) is also comparable to recent prevalence rates from Latvia (68%) [27]. Our finding has important implications for public health policy in Estonia. To date, many practitioners had believed that Estonia has been relatively spared from the global obesity epidemic, and its attentions should be turned elsewhere. Our data presents contrary evidence, by which the problem of obesity is as big in Estonia as in other Western countries, if not bigger, and hence deserves increasing attention in the public health policy sphere.

### Correlations between HMW adiponectin and metabolic risk factors

As expected, we found HMW adiponectin to correlate positively with HDL cholesterol and negatively with obesity, insulin resistance, triglycerides and fasting glucose for both genders. For women only, we found HMW adiponectin to correlate negatively with 2 h glucose in the OGTT, and with systolic and diastolic blood pressure. These results are similar to the outcomes of Andreasson *et al*. who observed gender-related differences in the association between adiponectin and cardiovascular risk factors: total adiponectin was associated with blood lipids in both men and women, but adiponectin was associated with glucose homeostasis more in women than in men [30]. Our study adds to this literature by describing this relationship in terms of HMW adiponectin, instead of total adiponectin. In contrast, in KORA survey, total adiponectin correlated to various metabolic risk factors in a pattern that was similar in both sexes [31]. We found HMW adiponectin to correlate to blood pressure exclusively in women. Clinical and experimental studies indicate a causal relationship between low adiponectin and hypertension [32], but it is still unclear to what extent this applies to men as well as women. On the one hand, a recent Japanese study showed that serum HMW adiponectin concentrations were inversely associated with blood pressure in the general male population [10]. In contrast, earlier studies on Japanese [9], Chinese [11] and Thai subjects [12] found no correlation between HMW adiponectin and systolic blood pressure. Significant differences exist in HMW adiponectin levels between difference ethnic groups [33], and to date most of the work has been carried out on Asian populations. Our study adds information about the associations between HMW adiponectin and blood pressure specifically for Caucasians.

### Comparison of HMW adiponectin and metabolic risk factors between metabolically healthy and unhealthy subjects

We found some interesting gender difference when comparing the association between HMW adiponectin and metabolic risk factors between metabolically healthy and unhealthy subjects. In metabolically healthy men, no correlations were found between HMW adiponectin and metabolic risk factors, with the exception of one risk factor: systolic blood pressure. Surprisingly, this relationship was not negative but positive. Our metabolically healthy group of men (n = 52) had a mean systolic blood pressure of 119.8± SD 7.2 mmHg (range 88.0–129.0). To the contrary, a recent study in Japan found higher adiponectin to correlate with lower systolic and diastolic blood pressure in normotensive people [34]. Associations between HMW adiponectin and cardiovascular disease are complex and influenced by several conditions and factors (chronic heart disease, chronic kidney disease, cachexia, underlying disease state) [35], [36]. Therefore this unexpected positive association between HMW adiponectin and systolic blood pressure in metabolically healthy men may be affected by other underlying factors.

Differentially from metabolically healthy men, we found HMW adiponectin to correlate with HDL cholesterol, triglycerides and insulin resistance among the metabolically healthy female subgroup. Comparisons of metabolically healthy and unhealthy subjects showed, as expected, that metabolically unhealthy subjects were significantly older, had significantly higher BMI, waist circumference and HOMA-IR compared with metabolically healthy subjects in both genders. In addition, we found that for women only, metabolically healthy subjects had significantly higher HMW adiponectin levels compared with metabolically unhealthy subjects. This result is in line with our previous finding that HMW adiponectin correlates with metabolic risk factors in the metabolically healthy women group, but not in metabolically healthy men. To the best of our knowledge, previous studies have not described such gender differences in the relationship between HMW adiponectin levels and metabolic risk factors between metabolically healthy and unhealthy subjects. Whether and how this gender-specific difference influences the pathophysiologial processes through which HMW adiponectin influences health and disease is worthy of further study. As a start, our data suggests that associations between HMW adiponectin and metabolic risk factors are complex and influenced by gender. It is known that testosterone reduces selectively the HMW form of adiponectin which might contribute to the gender difference of adiponectin levels [37]. A recent study from Spain showed that adiponectin is related to free androgen index and sex hormone binding globulin levels in adolescents after adjusting for BMI and fat mass, suggesting an association between adiponectin and androgen bioavailability [38]. On the other hand, serum adiponectin level was positively correlated with testosterone concentration in aging (50–85 years) men but not in women [39]. Thus, other factors beside testosterone might also play a role in gender difference of adiponectin levels [40]. We therefore encourage future studies to take a comprehensive approach to data collection and analysis, so to best develop our understanding of this biochemical system that influences the pathogenesis of today's most common diseases.

### Comparison of HMW adiponectin levels between metabolically healthy Vs unhealthy overweight/obese subjects

A subset of obese subjects seems to be protected from obesity-related cardiovascular and metabolic abnormalities. Metabolically normal obesity describes individuals with a body mass index ≥30 kg/m^2^, but who do not have any overt cardiometabolic diseases such as type 2 diabetes mellitus, dyslipidaemia and hypertension. Components of the metabolic syndrome, such as inflammatory markers and insulin sensitivity, have also been used to categorize subjects as metabolically healthy or unhealthy. However, it is not clear whether this metabolically healthy obese phenotype is just an early stage in the disease process that will ultimately progress to metabolically unhealthy obesity, or whether metabolically healthy obese people are relatively protected from developing the co-morbidities associated with obesity for the remainder of their lives [14]. Within our overweight sample, the metabolically healthy subgroup was approximately 10 years younger than the metabolically unhealthy subgroup. It may still be that we are seeing two different snapshots of the same process in time, whereby metabolically healthy subjects tend to transition to becoming increasingly metabolically unhealthy over time. However, in contrast to this hypothesis, our obese sample showed similar age distributions between the metabolically healthy and unhealthy subjects. Therefore our data does not allow us to refute either hypothesis. Future longitudinal studies could establish whether these metabolically healthy and unhealthy phenotypes are distinct only in time, or also in nature.

While no established criteria exist for the definition of metabolically healthy obese individuals [41], previous studies have reported that these individuals constitute approximately 6−35% of obese subjects depending on the definition [14]. We defined metabolically healthy status by strict criteria (subjects without impaired glucose regulation, dyslipidaemia, and hypertension and insulin resistance) and found that 12% of obese subjects were metabolically healthy. Further analysis showed that in both genders, these metabolically healthy obese subjects had HMW adiponectin levels comparable to normal weight subjects. These results are in line with outcomes of recent study from Poland, which also showed no differences in HMW adiponectin concentrations between overweight or obese but metabolically healthy women, and normal weight controls [18]. Our data adds useful information in that this phenomenon also exists for obese but metabolically healthy Caucasian men.

We found that among women with BMI ≥25 kg/m^2^, HMW adiponectin levels were higher in metabolically healthy women compared to metabolically unhealthy women. We did not find a comparable difference in men. However, among obese men, metabolically healthy men had significantly higher HMW adiponectin levels compared with metabolically unhealthy men. While we cannot explain this discrepancy, we are aware that the pathogenesis of metabolic disease is complex and includes various factors excluded from our study. Kim *et al* has shown that mice lacking leptine while overexpressing adiponectin had significantly higher levels of adipose tissue than they *ob/ob* littermates, but an improved metabolic profile [42]. Therefore, there are many other adipocytokines besides adiponectin which modify the pathogenesis of metabolic disorders. Moreover, they are likely to influence each other's expression and production [3]–[5]. We measured only one adipocytokine isoform (HMW adiponectin) and hence only looked at one aspect of the complex association between adipose tissue dysfunction and metabolic risk factors.

Our study also had the following **limitations:** the possible complex effect of other underlying diseases (and medications used for other diseases) on HMW adiponectin levels could not be excluded; our metabolically healthy obese subgroup was very small; the metabolically healthy obese phenotype definitions have not been standardized; and BMI as a measure of obesity has limitations because it cannot distinguish between fat tissue and lean tissue. Despite these limitations, our study had also several **strengths:** our population-based approach; measuring not total but HMW adiponectin; strict criteria to define metabolically healthy subgroups; and impaired glucose regulation diagnosed by a comprehensive oral glucose tolerance test.

**To conclude,** the prevalence of obesity in Estonian adult population was 32%. This is significantly higher than previously estimated, but comparable with recently reported data from other European countries. This finding elevates the relative importance of obesity as a public health hazard in Estonia.

In metabolically healthy women, HMW adiponectin levels were associated with various metabolic risk factors. This association was not present in metabolically healthy men. 12% of obese subjects were metabolically healthy. This phenotype was characterised by HMW adiponectin levels similar to normal weight subjects. These findings take us one step closer in understanding in more detail how this important biochemical molecule works in Caucasians, in contributing to the pathogenesis of today's most prevalent diseases. However, further prospective population-based studies are needed to investigate the mechanisms of adiponectin actions in order to answer these questions.
